# Analysis of the Gut Microbiota: An Emerging Source of Biomarkers for Immune Checkpoint Blockade Therapy in Non-Small Cell Lung Cancer

**DOI:** 10.3390/cancers13112514

**Published:** 2021-05-21

**Authors:** Feiyu Zhang, Macarena Ferrero, Ning Dong, Giuseppe D’Auria, Mariana Reyes-Prieto, Alejandro Herreros-Pomares, Silvia Calabuig-Fariñas, Elena Duréndez, Francisco Aparisi, Ana Blasco, Clara García, Carlos Camps, Eloisa Jantus-Lewintre, Rafael Sirera

**Affiliations:** 1 Molecular Oncology Laboratory, Fundación Investigación, Hospital General Universitario de Valencia, 46014 Valencia, Spain; zfei@alumni.uv.es (F.Z.); ferrero_mac@gva.es (M.F.); dongning@alumni.uv.es (N.D.); alherpo@etsiamn.upv.es (A.H.-P.); calabuix_sil@gva.es (S.C.-F.); medusae@posgrado.upv.es (E.D.); camps_car@gva.es (C.C.); 2 Unidad Mixta TRIAL, Centro Investigación Príncipe Felipe—Fundación Investigación, Hospital General Universitario de Valencia, 46014 Valencia, Spain; blasco_ana@gva.es; 3 Centro de Investigación Biomédica en Red Cáncer, CIBERONC, 28029 Madrid, Spain; 4 Sequencing and Bioinformatics Service, Fundació per al Foment de la Investigació Sanitària i Biomèdica de la Comunitat Valenciana, FISABIO, 46020 Valencia, Spain; dauria_giu@gva.es (G.D.); reyes_ber@gva.es (M.R.-P.); 5 Centro de Investigación Biomédica en Red Epidemiología y Salud Pública, CIBERESP, 28029 Madrid, Spain; 6 Evolutionary Genetics, Institute for Integrative Systems Biology, University of Valencia and Spanish Research Council, 46980 Valencia, Spain; 7 Department of Pathology, Universitat de València, 46010 Valencia, Spain; 8 Department of Medical Oncology, Hospital General de Requena, 46340 Valencia, Spain; aparisi_fraapa@gva.es; 9 Department of Medical Oncology, Hospital General Universitario de Valencia, 46014 Valencia, Spain; garcia_clagon@gva.es; 10 Department of Medicine, Universitat de València, 46010 Valencia, Spain; 11 Department of Biotechnology, Universitat Politècnica de València, 46022 Valencia, Spain

**Keywords:** gut microbiota, biomarker, non-small cell lung cancer, immune checkpoint blockade, immunotherapy, next-generation sequencing

## Abstract

**Simple Summary:**

The immune checkpoint blockade (ICB), and concretely the blockade of the PD1/PDL1 axis, has opened up a new standard of treatment for non-small cell lung cancer (NSCLC). However, despite substantial advances in clinical care, many patients still remain refractory to these therapies. Biomarkers such as PD-L1 expression and tumor mutational burden have been associated with ICB efficacy, but the mechanisms underlying variable responses are not yet fully understood. Recently, the differential composition of the gut microbiota was studied as one of the variables accounting for interpatient heterogeneity in ICB responses. To better understand the potential role of the gut microbiota as a biomarker for immunotherapy, we prospectively collected microbiota samples from advanced NSCLC patients starting treatment with ICB. The identification of certain bacteria genera associated with clinical outcomes to ICB in NSCLC may provide novel potential predictive and prognostic biomarkers useful for patient selection and therapy optimization.

**Abstract:**

Background: The human gut harbors around 10^13^–10^14^ microorganisms, collectively referred to as gut microbiota. Recent studies have found that the gut microbiota may have an impact on the interaction between immune regulation and anti-cancer immunotherapies. Methods: In order to characterize the diversity and composition of commensal microbiota and its relationship with response to immune checkpoint blockade (ICB), 16S ribosomal DNA (rDNA) sequencing was performed on 69 stool samples from advanced non-small cell lung cancer (NSCLC) patients prior to treatment with ICB. Results: The use of antibiotics and ICB-related skin toxicity were significantly associated with reduced gut microbiota diversity. However, antibiotics (ATB) usage was not related to low ICB efficacy. *Phascolarctobacterium* was enriched in patients with clinical benefit and correlated with prolonged progression-free survival, whereas *Dialister* was more represented in patients with progressive disease, and its higher relative abundance was associated with reduced progression-free survival and overall survival, with independent prognostic value in multivariate analysis. Conclusions: Our results corroborate the relation between the baseline gut microbiota composition and ICB clinical outcomes in advanced NSCLC patients, and provide novel potential predictive and prognostic biomarkers for immunotherapy in NSCLC.

## 1. Introduction

Lung cancer is one of the most lethal cancers worldwide [1,2]. Non-small cell lung cancer (NSCLC) constitutes approximately 85% of all lung cancers and includes adenocarcinoma, squamous cell carcinoma (SCC), and large cell carcinoma as the main histological subtypes [3,4]. The treatment of NSCLC has changed in recent years with the initial success of immunotherapy, especially that based on immune checkpoint blockade (ICB) with monoclonal antibodies against cytotoxic T lymphocyte-associated protein 4 (CTLA-4) or programmed cell death protein 1 (PD-1) and its ligand (PD-L1). The PD-1/PD-L1 axis has been demonstrated to influence the balance between tumor immune surveillance and immune resistance. In this sense, elevated PD-L1 expression on tumor cells results in T cell exhaustion, thereby attenuating tumor-specific immunity and promoting tumor progression [5]. Anti-PD-1 (nivolumab, pembrolizumab, cemiplimab) and anti-PD-L1 antibodies (atezolizumab, avelumab, durvalumab) are on the list of approved agents for different types of cancer, including NSCLC [6,7,8]. Clinical responses to treatment with ICB are usually durable, and the overall response rate in advanced NSCLC is around 15–30% [9,10,11,12,13]. However, despite substantial clinical advance, many patients do not benefit from these therapies and, therefore, identification of biomarkers to expand therapeutic efficacy of ICB is a priority. The expression of PD-L1 is currently the most extended and widely employed predictive biomarker for both PD-1 and PD-L1 blockade in NSCLC [14]. However, the use of this biomarker still presents unresolved issues, including temporal and spatial expression heterogeneity as well as variable quantitative scores and cutoffs within and across tumor types, which may exclude a considerable number of responders [15,16]. More recently, tumor mutational burden (TMB), defined as the number of non-synonymous mutations per coding area of tumor genome, has become a useful predictive marker for ICB effectiveness, regardless of PD-L1 expression, in different types of solid tumors, including NSCLC [17,18]. Nonetheless, some caveats, such as unclear thresholds for positive results along studies and tumor histologies, limited data providing significant correlation between higher TMB and improved overall survival, and the fact that not all patients with high TMB derive benefit from ICB, indicate that this biomarker alone is not the perfect predictor for immunotherapy [19]. Moreover, TMB determination by next-generation sequencing is often challenging because of the difficulty to obtain tumor tissue in many of the cases. In this sense, assessment of TMB in liquid biopsies and cytological specimens represent promising alternatives. Interestingly, it has been demonstrated that performing TMB evaluation on cytological samples with TMB values comparable to those obtained in histologically matched samples is technically feasible [20]. However, these findings need to be confirmed in large-scale studies and are still under investigation to accurately direct therapeutic decisions.

On the other hand, immune-related adverse events (irAEs) also remain an important therapeutic problem for immunotherapy usage. A systematic review of 5744 NSCLC patients treated with anti-PD-1/PD-L1 reports an overall adverse events incidence of 64% for anti-PD-1 and 66% for anti-PD-L1 agents [21]. IrAEs can involve any organ or system, and endocrine, dermatological, and gastrointestinal toxicities are the most common irAEs associated with anti-PD-1/PD-L1 in NSCLC patients [22]. Currently, predictive biomarkers for these irAEs to facilitate the use of ICB in the clinical setting are not available. Taking into consideration the evidence, identification of new reliable biomarkers to guide patient selection and provide indications of efficacy and/or toxicity for ICB therapies is of utmost importance.

Most recent research has been focused on the potential use of the gut microbiota as a biomarker for immunotherapy response. The human gut houses more than 10^13^ microorganisms [23], and the collection of these microorganisms is commonly termed gut microbiota. Abnormal composition of the gut microbiota has been proven to be related to many diseases, including inflammatory bowel disease and metabolic diseases [24,25]. Recently, there is an emerging idea of the systemic influence of the gut microbiota, and the “gut–lung” axis hypothesis supports the importance of a healthy gut microbiota to produce effective immune responses in the lung. Moreover, the impact of the microbiota on cancer and cancer treatment is an emerging area of great interest [26,27]. In the era of novel immune-modulating agents, differential composition of the gut microbiota has been studied as one of the variables accounting for interpatient heterogeneity in ICB responses [28,29,30,31,32]. Additionally, lung cancer patients are frequently treated with antibiotics (ATB) and this intervention could modify or unbalance the gut microbiota. In this regard, the impact of ATB on the clinical outcomes of patients treated with ICB should be a priority area of research [33,34].

Preclinical mouse models have highlighted that the therapeutic efficacy of immunotherapy is strongly dependent on the gut microbiota. In this scenario, *Bacteroides fragilis* has been confirmed to be related to higher anti-CTLA-4 treatment efficiency by regulating the function of dendritic cells and differentiation of T helper cells [35]. *Bifidobacterium spp*. and *Akkermansia muciniphila* have been associated with efficacy of anti-PD-1/PD-L1 treatment by increasing the function of dendritic cells, and enhancing activation and recruitment of CD4+ and CD8+ T cells into the tumor microenvironment [29,30,36]. A defined combination of 11 microbial strains has been associated with high colon interferon-γ (IFN-γ) production by CD8+ T cells and correlated with enhanced therapeutic efficacy of ICB in mouse tumor models [37]. Moreover, microbiota-derived end product metabolites from dietary fermentation, such as short-chain fatty acids (SCFAs), have been confirmed to influence the ICB clinical outcome by regulating the immune response [38,39]. In addition to the role in shaping systemic immune responses to ICB, the gut microbiota may also influence the emergence of irAEs, and its modification has been demonstrated to ameliorate certain adverse events, particularly colitis [28,40,41].

To better understand the potential role of the gut microbiota as a biomarker for immunotherapy, we sequenced the 16S ribosomal DNA (rDNA) gene in stool samples from 69 advanced NSCLC patients starting ICB. We compared gut microbiota diversity and composition among patients with clinical benefit and patients experiencing progression disease, and evaluated the correlation of specific taxonomic genera with survival indicators. The identification of determined genera, concretely *Phascolarctobacterium* and *Dialister*, associated with clinical outcomes of advanced NSCLC patients undergoing ICB might provide novel potential predictive/prognostic biomarkers useful for patient selection and therapy optimization. In addition, we studied the gut microbiota composition in patients who developed immune-related toxicities, observing that *Bacteroides dorei* was enriched in samples from patients with skin toxicity, whereas bacteria from the *Firmicutes* phylum and *Bacteroides vulgatus* were predominant in patients who did not experience dermatological toxicity. Importantly, our results did not reveal any relationship between ATB usage and diminished response to or survival from ICB immunotherapy.

## 2. Materials and Methods

### 2.1. Patient Cohort

A total of 69 patients with advanced NSCLC were included in this prospective study. Eligible patients did not harbor epidermal growth factor receptor (*EGFR*) and anaplastic lymphoma kinase (*ALK*) gene mutations within the tumors, and did not receive previous targeted therapy. Based on the absolute number of white blood cells (leukocytes, lymphocytes, neutrophils, and monocytes), determined by routine blood testing, the vast majority of patients presented an adequate immune status to receive immunotherapy. All patients were treated with ICB at the General University Hospital Consortium of Valencia between November 2017 and June 2019. The patients received ICB monotherapy, including nivolumab (3 mg/kg, once every 2 weeks), pembrolizumab (2 mg/kg, once every 3 weeks), or atezolizumab (1200 mg, once every 3 weeks). Evaluation of ICB treatment efficacy was done according to Response Evaluation Criteria in Solid Tumors version 1.1 (*RECIST 1.1*) [42]. The patients were classified into two groups: clinical benefit (CB) group (complete response, partial response, and stable disease lasting more than 6 months) and progression disease (PD) group (progressive disease and stable disease lasting less than 6 months).

This study was conducted in accordance with the Declaration of Helsinki for research involving human subjects. All patients enrolled signed a voluntary informed consent for fecal sample collection and study under the approved protocols of the Clinical Research Ethics Committee of the present institution (General University Hospital Consortium of Valencia).

### 2.2. Sample Collection

Stool samples from 69 NSCLC patients were collected with EasySampler-Complete Stool Collection Kit (ALPCO, Salem, NH, USA). All patients were provided with stool sample containers and completed their depositions at home within 3 days before ICB treatment initiation. The samples were brought to the laboratory within 6 h after collection and stored at −80 °C until processing.

### 2.3. DNA Extraction and 16S rDNA Gene Sequencing

QIAamp Fast DNA Stool Mini Kit (Qiagen, Hilden, Germany) was used for genomic DNA extraction from stool samples, according to the manufacturer’s protocol.

Isolated DNA was sent to the Sequencing and Bioinformatics Service of the Foundation for the Promotion of Health and Biomedical Research of Valencia Region (FISABIO) for sequencing. In more detail, the 16S rDNA gene V3-V4 hypervariable region was amplified using the primers described by Klindworth et al. [43] and then processed using 16S Metagenomic Sequencing Library Preparation Illumina protocol (Cod. 15044223 Rev. A, Illumina, Inc., San Diego, CA, USA). The full-length primer sequences including Illumina adaptors were as follows: forward: TCGTCGGCAGCGTCAGATGTGTATAAGAGACAGCCTACGGGNGGCWGCAG, and reverse: GTCTCGTGGGCTCGGAGATGTGTATAAGAGACAGGACTACHVGGGTATCTAATCC. Microbial genomic DNA (5 ng/μL in 10 mM Tris, pH 8.5) was used to start the polymerase chain reaction (PCR) protocol. After 16S rDNA gene amplification, the multiplexing step was performed using Nextera XT Index Kit (FC-131-2001). A volume of 1 μL of the PCR product was run on a Bioanalyzer DNA 1000 chip to verify the size, being the expected size on a Bioanalyzer trace of ~550 bp. After size verification the libraries were sequenced using a 2 × 300 pb paired-end run (MiSeq Reagent kit v3 (MS-102-3003)) on a MiSeq sequencer, according to the manufacturer’s instructions (Illumina).

### 2.4. Bioinformatic Analysis and Taxonomic Annotation

Amplicon sequences were processed using the QIIME2 pipeline [44]. The DADA2 pipeline was employed for denoising, paired-end joining, and chimera depletion from paired-end data [45]. Taxonomic affiliations were assigned using the Naive Bayesian classifier integrated in QIIME2 plugins. The SILVA release 132 database was used for taxonomic assignation [46]. The taxonomic composition of the gut microbiota was generated by different levels: kingdom, phylum, class, order, family, genus, and species. Alpha-diversity was estimated by Chao 1 richness, Shannon, or inverse Simpson diversity indices, according to the proportion of taxa counts within the QIIME2 pipeline.

### 2.5. Statistical Analysis

Statistical analyses were performed using SPSS 24.0 software (IBM Corp., Armonk, NY, USA), GraphPad Prism 6.0 (GraphPad Software Inc., San Diego, CA, USA), Microsoft Office Excel 2007 (Microsoft Inc., Redmond, WA, USA) or R software v.4.0.3 (R Foundation for Statistical Computing, Vienna, Austria). Chi-squared test and nonparametric tests (Mann–Whitney *U* or Kruskal–Wallis) were applied to evaluate associations between patient clinico-pathological characteristics and microbiota composition. Permutational multivariate analysis of variance (PERMANOVA) was used to compare compositional differences between groups. Linear discriminant analysis (LDA) effect size (LEfSe) was performed to study the differential abundance analysis of gut microbiota composition [47]. Progression-free survival (PFS) and overall survival (OS) estimations were performed by the Kaplan–Meier method and compared with the log-rank test. Hazard ratios and 95% confidence intervals were calculated by the Cox regression model. Multivariate Cox regression analysis was conducted to further identify independent prognostic factors associated with PFS and OS. All results were accepted statistically if *p*-value < 0.05.

## 3. Results

### 3.1. Baseline and Sequencing Characteristics

The clinico-pathological characteristics of the 69 advanced NSCLC patients included in this study are shown in Table S1. The median age in this cohort was 67, 23.2% of the patients were prescribed ATB within 3 months before ICB treatment, 66.7% presented PD-L1- positive expression (TPS ≥ 1%), and 53.6% were treated with ICB in first-line setting. The median follow-up time was 14.3 months, and according to response, 33 (47.8%) patients were classified in the clinical benefit (CB) group and 36 (52.2%) in the progression disease (PD) group. Correlation analysis revealed that gender, body mass index, and PD-L1 expression were clinical factors associated with response to ICB in this cohort (Table 1). Univariate Cox regression analysis evidenced that PFS was significantly shorter in women, patients with poor Eastern cooperative oncology group (ECOG) performance status, and patients with low PD-L1 expression, whereas prolonged OS was observed in patients with high body mass index (Table S2).

**Figure 1:**
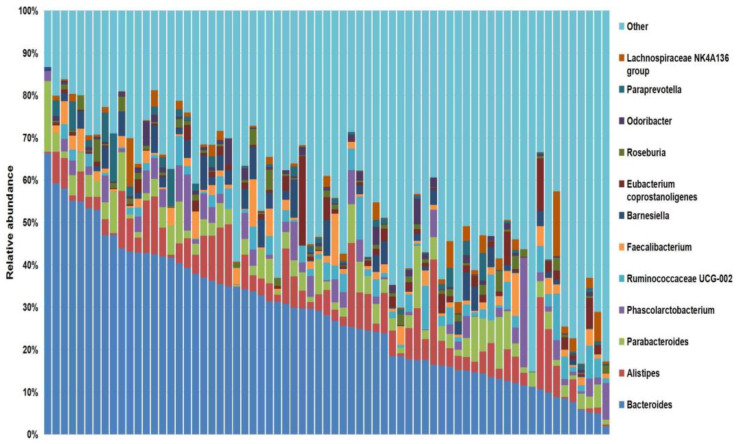
Stacked bar plot of the phylogenetic composition of the most common taxa at the genus level. *Bacteroides*, in dark blue, was the most frequent genera identified within the cohort.

Barcoded 16S rDNA amplicon sequencing of the 69 stool samples yielded a total of 10,553,396 reads and 7,435,908 (70.5%) effective reads after filtering, with a median of 106,425 reads per sample (range 39,672–395,132). An overview of the sequencing information and microbiota diversity indices, including Chao 1 (range 42–274), Shannon (range 1.78–4.30), and inverse Simpson (range 3.32–42.93), of 69 advanced NSCLC patients in our cohort are shown in Table S3. A total of 851 taxa were obtained, and at the genus level, *Bacteroides* was the most frequent genus among the 357 genera detected in this study (median relative abundance: 28%) (Figure 1).

### 3.2. Impact of ATB Usage on the Diversity and Composition of the Gut Microbiota

**Figure 2:**
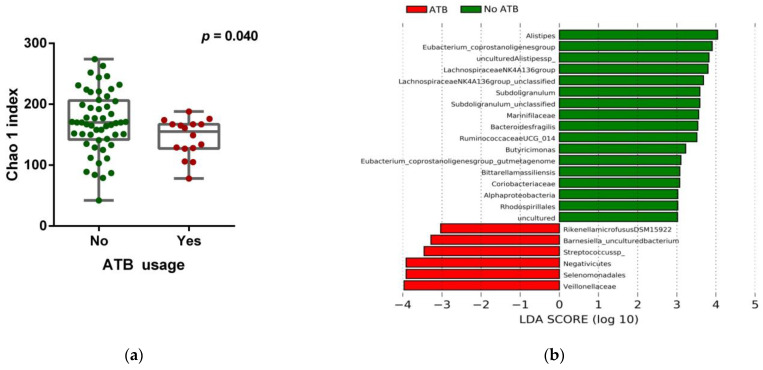

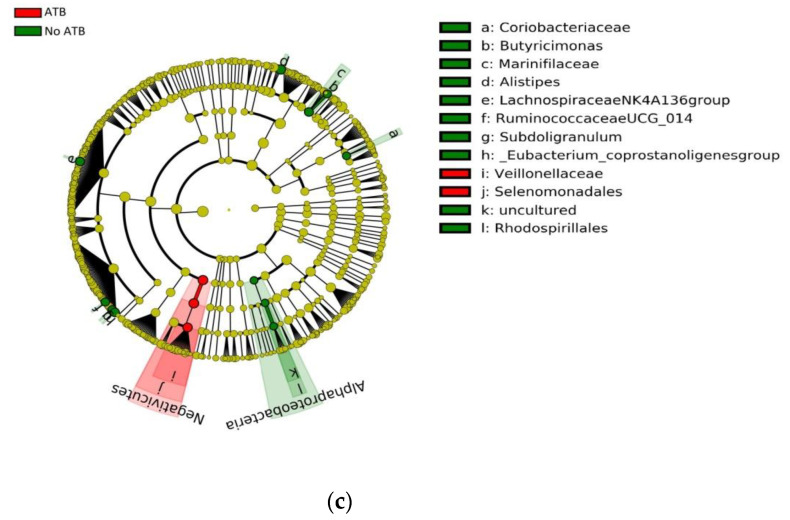
Gut microbiota composition and ATB usage. (**a**) Chao 1 index score of the gut microbiota in patients stratified according to ATB usage, by the Mann–Whitney *U* rank sum test. Error bars represent the distribution of alpha-diversity scores. (**b**) Multi-level differential abundance analysis using linear discriminant analysis (LDA) effect size (LEfSe) of patients stratified according to ATB usage. (**c**) Taxonomic cladogram from the LEfSe analysis showing differences in fecal taxa in ATB and non-ATB groups. Dot size is proportional to the abundance of the taxon. Green legends represent patients who did not receive ATB treatment, and red legends the patients who were prescribed ATB within 3 months before ICB treatment. ATB: antibiotics. LDA score > 3.0; *p* < 0.05.

To study the complexity of the microbiota community structure within the samples, we determined alpha-diversity scores for all patients (Table S3) and found that patients receiving ATB within 3 months before ICB treatment (16/69, 23.2%) significantly exhibited lower Chao 1 index (*p* = 0.040) (Figure 2a and Table S4), indicating a certain grade of dysbiosis. Compositional analysis by linear discriminant analysis of effect size (LEfSe) revealed that feces samples of patients without ATB treatment were enriched with the genus *Alistipes*, whereas the family *Veillonellaceae* was predominant in the ATB group, among other taxa (Figure 2b,c).

To explore whether dysbiosis associated with ATB use could influence resistance to PD-1 blockade, we also compared the therapeutic efficacy of ICB in patients treated with ATB and those not treated with ATB. No significant differences were observed between CB and PD groups based on ATB usage (Table 1), indicating that response to ICB was not impacted by the use of ATB in our cohort. Regarding survival indicators, we neither found statistically significant differences in PFS and OS between ATB and non-ATB groups, suggesting that ATB usage was not associated with poorer prognosis in the cohort (Table S2).

### 3.3. Correlation of the Gut Microbiota with Immune-Related Adverse Events

A total of 26/69 (37.7%) patients experienced irAEs at any grade. The different types of developed irAEs included endocrine (13/69, 18.8%), hepatic (10/69, 14.5%), gastrointestinal (8/69, 11.6%), dermatological (7/69, 10.1%), renal (6/69, 8.7%), and pulmonary (pneumonitis) toxicities (3/69, 4.3%) (Table S1). Interestingly, development of irAEs predicted clinical benefit and was correlated with improved PFS and OS in our cohort (Figure S1).

**Figure 3:**
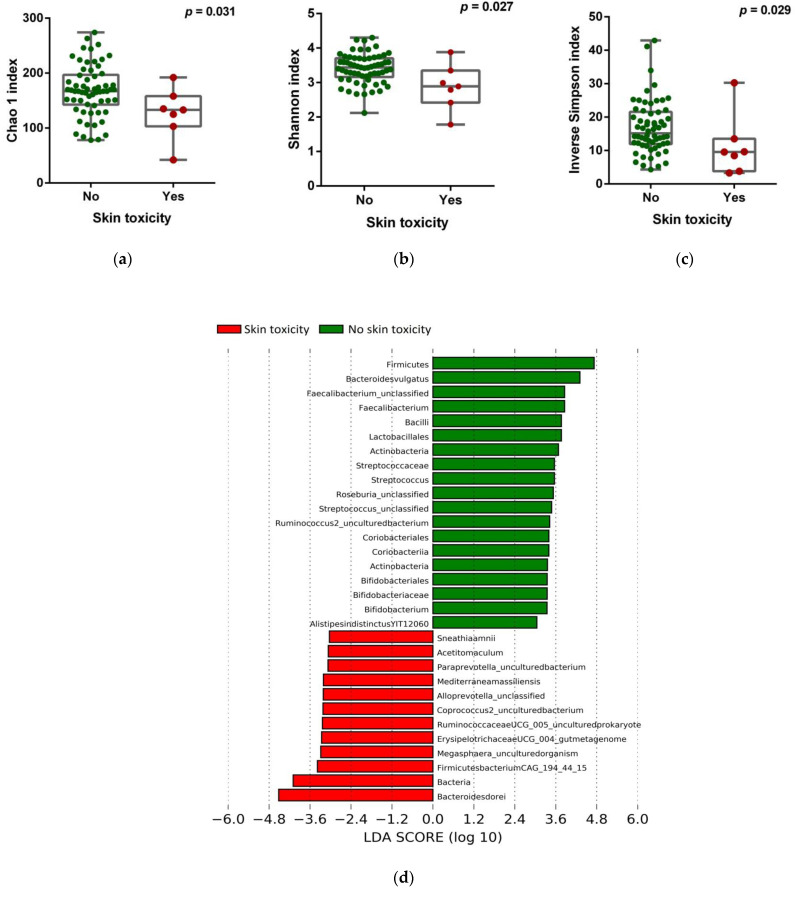

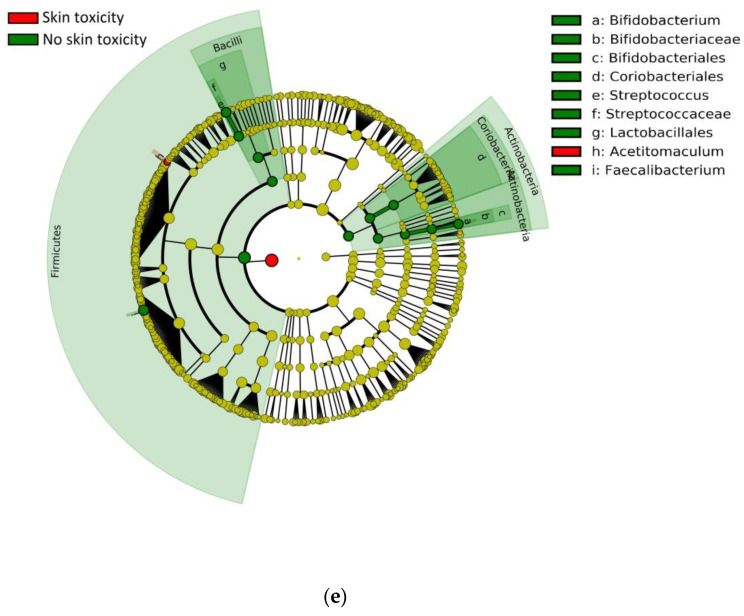
Gut microbiota composition and ICB-related skin toxicity. (**a**) Chao 1, (**b**) Shannon, and (**c**) inverse Simpson indices scores of the gut microbiota in patients stratified according to the presence/absence of skin toxicity by Mann–Whitney *U* rank sum test. Error bars represent the distribution of alpha-diversity scores. (**d**) Multi-level differential abundance analysis using linear discriminant analysis (LDA) effect size (LEfSe) in patients stratified according to skin toxicity. (**e**) Taxonomic cladogram stratified by skin toxicity. Dot size is proportional to the abundance of the taxon. Green legends represent the patients without skin toxicity, and red legends represent the patients with skin toxicity associated with ICB treatment. LDA score > 3.0; *p* < 0.05.

To investigate the possible influence of the gut microbiota on irAEs, we first analyzed the relationship between alpha-diversity indices and different types of irAEs. According to severity, no significant differences were observed in bacterial diversity between patients who experienced relevant clinical toxicities (≥grade 2) and patients with non-severe toxicities (grade 1 or absent, Figure S2). However, according to toxicity types, it was found that patients showing ICB-related skin toxicity exhibited a significant decrease in alpha-diversity indices compared to those without skin toxicity (Chao 1 index, *p* = 0.031; Shannon index, *p* = 0.027; inverse Simpson index, *p* = 0.029, respectively, Figure 3a–c). No significant differences in bacterial diversity were observed for the other types of irAEs (Figure S2). Compositional analysis by LEfSe demonstrated differential abundances of bacteria in the gut microbiota of patients with or without skin toxicity. Concretely, *Bacteroides dorei* was enriched in samples from patients with skin toxicity, while bacteria from the *Firmicutes* phylum and *Bacteroides vulgatus* species were predominant in patients who did not experience dermatological toxicity (Figure 3d,e). These findings indicate that baseline microbiota diversity and composition are associated with the emergence of immune-related skin toxicity and might represent potential biomarkers of dermatological manifestations.

### 3.4. Gut Microbiota Composition and Response to ICB

To address a possible link between gut microbiota diversity and ICB clinical outcomes, we examined alpha-diversity indices in patients stratified according to ICB response. No significant differences were found in alpha-diversity between CB and PD patients, suggesting similarity in the compositional complexity of the gut microbiota among these two groups of patients (Figure S3). Moreover, to seek a possible impact of diversity on survival, we stratified patients into high versus low categories, based on the median of alpha-diversity indices. Again, no significant association of alpha-diversity with PFS and OS was found within the cohort, indicating a poor value of alpha-diversity as a prognostic factor (Figure S3).

**Figure 4:**
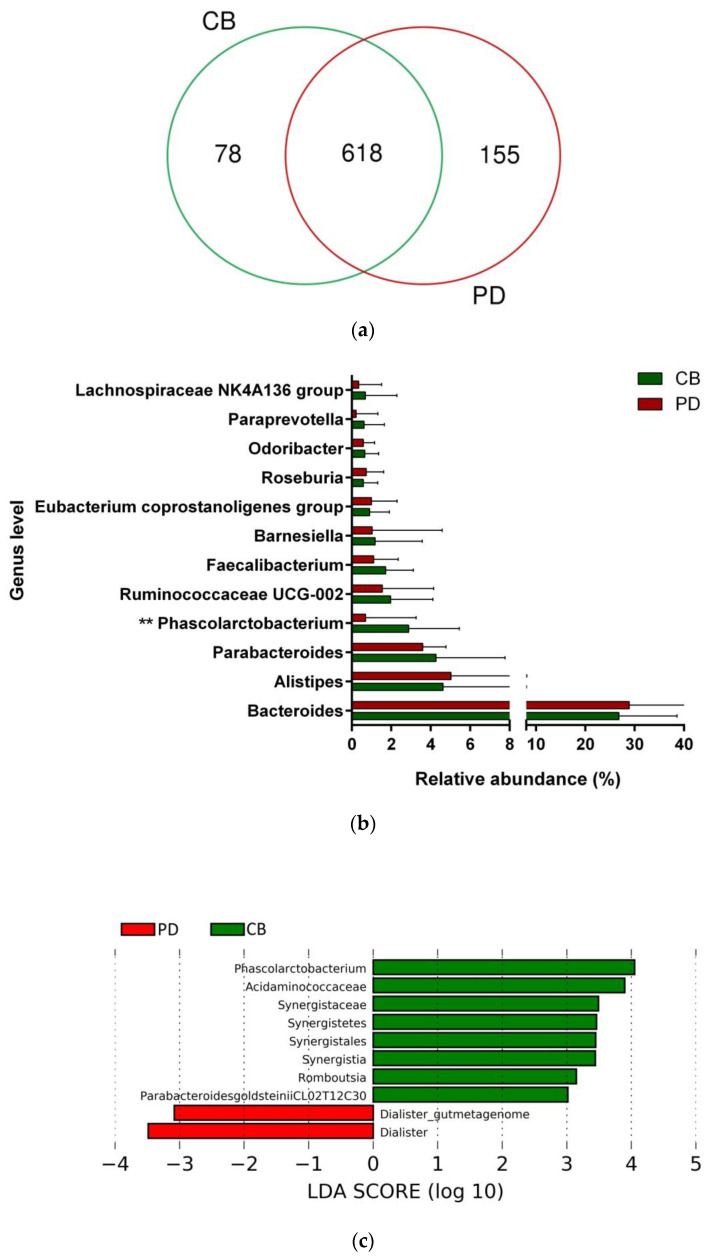

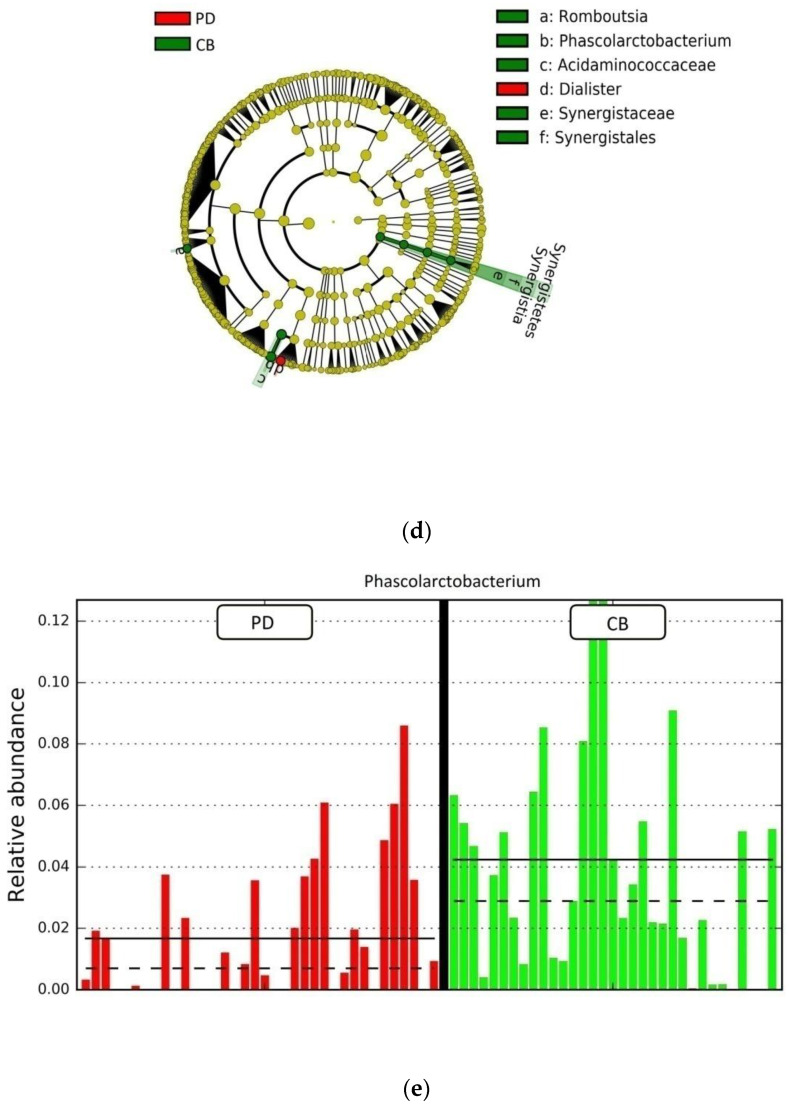

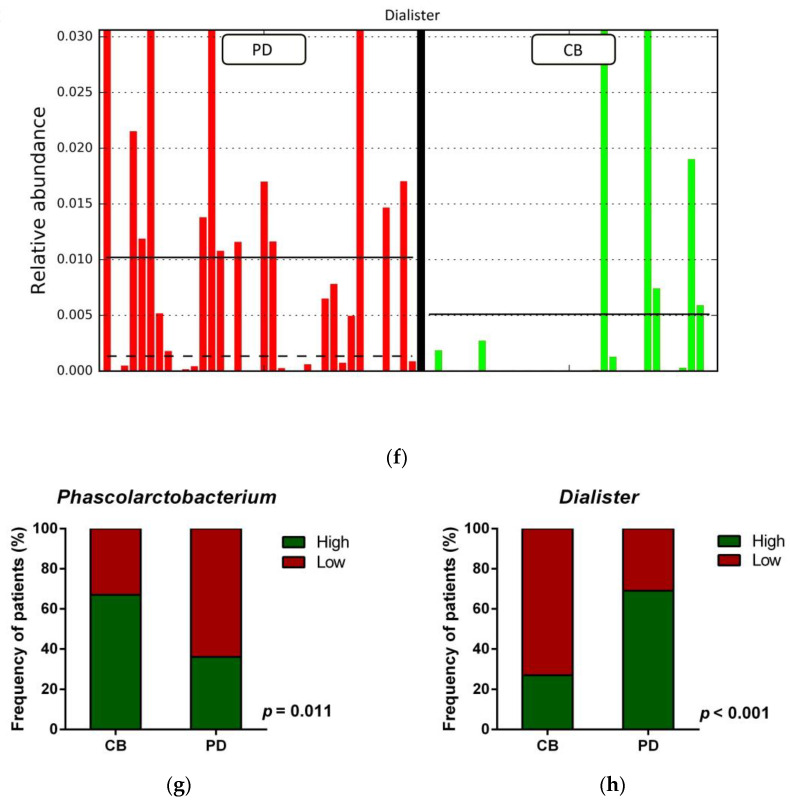
Compositional differences of the gut microbiota between CB and PD groups. (**a**) Venn diagram depicting shared and unique taxa for CB and PD groups; 618 taxa were identified as core microbiota (shared taxa). (**b**) Comparisons of the relative abundance at the genus level among CB (green) and PD (red) groups; ** *p* < 0.01. (**c**) Differential abundance analysis using linear discriminant analysis (LDA) effect size (LEfSe) in patients stratified according to response to ICB. (**d**) Taxonomic cladogram from the LEfSe analysis depicting differences in fecal taxa in CB and PD patients. Dot size is proportional to the abundance of the taxon. Green legends represent CB patients and red legends represent PD patients. (**e**) Differential abundance analysis using linear discriminant analysis (LDA) effect size (LEfSe) for the genera *Phascolarctobacterium* in PD and CB patients; the solid straight lines represent the plot subgroup relative abundance means; the dotted straight lines represent the plot subgroup relative abundance medians. (**f**) Differential abundance analysis using linear discriminant analysis (LDA) effect size (LEfSe) for the genus *Dialister* in PD and CB patients; the solid straight lines represent the plot subgroup relative abundance means; the dotted straight lines represent the plot subgroup relative abundance medians. (**g**) Frequency of patients with high versus low relative abundance of *Phascolarctobacterium* in their stool samples according to response to ICB (CB and PD groups). (**h**) Frequency of patients with high versus low relative abundance of *Dialister* in their fecal samples according to the response to ICB (CB and PD groups). The cutoff value is the median of relative abundance: 1.92% (*Phascolarctobacterium*), 0.01% (*Dialister*). CB: clinical benefit group; PD: progression disease group. LDA score > 3.0; *p* < 0.05.

To study whether differential composition and abundance within the gut microbiota could influence the patient clinical outcome to ICB, we analyzed the gut microbiota composition in CB and PD patients. To this aim, we compared the number of taxa between these two groups, observing that out of the 851 taxa obtained, 618 were shared among CB and PD patients (core microbiota), whereas 78 and 155 were exclusive to CB and PD groups, respectively (Figure 4a). However, these exclusive taxa were detected in a few patients and, therefore, the use of exclusive pools was avoided due to high individual variability (Figure S4). Accordingly, principal component analysis revealed no cluster formation in CB and PD patients, indicating that no specific cluster was associated with clinical outcome (Figure S5).

Focused on the core microbiota, we next characterized and compared the differential abundances of the most frequent genera detected in CB and PD patients. Among the most frequent genera identified (Figure 1), *Phascolarctobacterium* was significantly enriched in CB patients (Figure 4b). LEfSe analysis was next used to find further imbalanced microorganisms between CB and PD patients (LDA score > 3.0 and *p* < 0.05). Results showed that *Phascolarctobacterium* (LDA > 4.0), *Acidaminococcaceae*, *Synergistaceae*, *Synergistetes*, *Synergistales*, *Synergistia*, *Romboutsia*, and *Parabacteroidesgoldsteinii CL02T12C30* were enriched in CB patients, while *Dialister* and *Dialister gutmetagenome* were enriched in PD patients (Figure 4c–f). Among these bacteria, the *Phascolarctobacterium* and *Dialister* genera were found in at least 50% of patients. Interestingly, when stratifying patients into high versus low groups, based on the median relative abundance of these taxa, we found that high abundance of *Phascolarctobacterium* was detected in 67% (22/33) of patients experiencing clinical benefit, and 36% (13/36) of patients who progressed (*p* = 0.011, Figure 4g). Conversely, high abundance of *Dialister* was observed in 69% (25/36) of PD patients, but only in 27% (9/33) of CB patients (*p* < 0.001, Figure 4h). These results demonstrate the potential value of the genera *Phascolarctobacterium* and *Dialister* predicting response to ICB treatment.

### 3.5. Analysis of the Gut Microbiota as a Prognostic Marker

**Figure 5:**
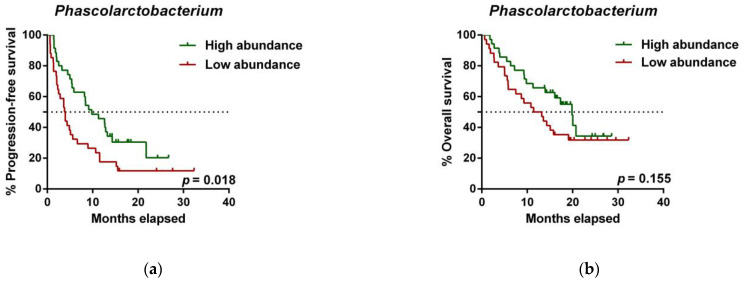

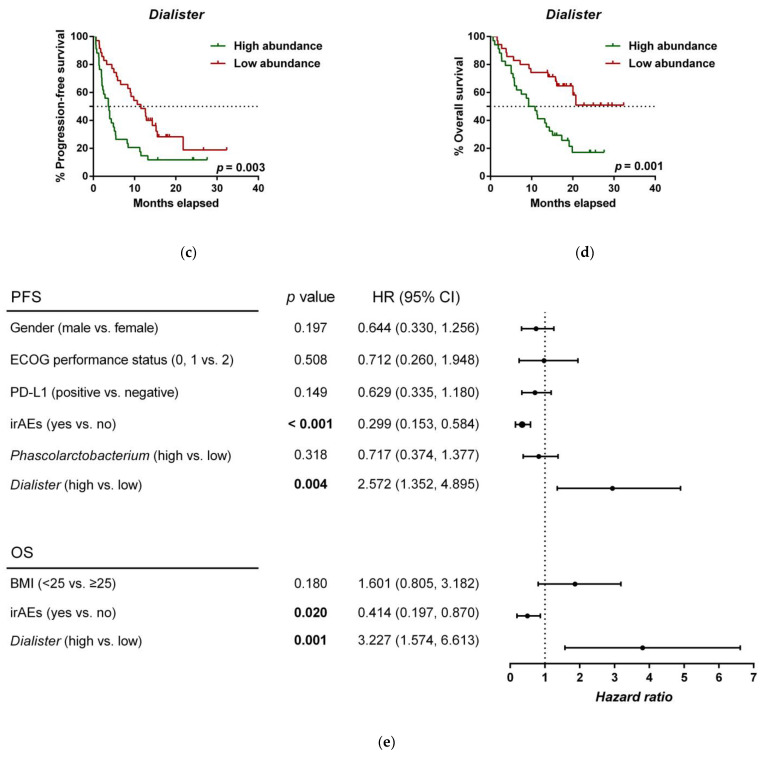
*Dialister* abundance and the presence of irAEs are independent prognostic factors for PFS and OS. (**a**) Kaplan–Meier plot of PFS in patients with high (green line, *n* = 35 (CB = 22, PD = 13), median PFS = 9.8 months) or low abundance of *Phascolarctobacterium* (red line, *n* = 34 (CB = 11, PD = 23), median PFS =3.8 months). (**b**) Kaplan–Meier plot of OS in patients with high (green line, *n* = 35 (CB = 22, PD = 13), median PFS = 19.9 months) or low abundance of *Phascolarctobacterium* (red line, *n* = 34 (CB = 11, PD = 23), median PFS = 11.4 months). (**c**) Kaplan–Meier plot of PFS in patients with high (green line, *n* = 34 (CB = 9, PD = 25), median PFS = 3.6 months) or low abundance of *Dialister* (red line, *n* = 35 (CB = 24, PD = 11), median PFS = 11.5 months). (**d**) Kaplan–Meier plot of OS in patients with high (green line, *n* = 34 (CB = 9, PD = 25), median PFS = 9.3 months) or low abundance of *Dialister* (red line, *n* = 35 (CB = 24, PD = 11), median PFS = not reached months). (**e**) Forest plot illustrating the results of multivariate analysis by the Cox proportional-hazard model. Statistical analysis was performed using the log-rank test. Bold *p*-values denote statistical significance at the *p* < 0.05 level. Cutoff values correspond to the median relative abundance: 1.92% (*Phascolarctobacterium*), 0.01% (*Dialister*). ECOG: Eastern cooperative oncology group; irAEs: immune-related adverse events; BMI: body mass index; PFS: progression-free survival; OS: overall survival; HR: hazard ratio; CI: confidence interval.

To further investigate the prognostic value of predictive bacteria, we performed survival analysis. Patients with high abundance of *Phascolarctobacterium* exhibited significantly prolonged PFS (median PFS 9.8 vs. 3.8 months, hazard ratio (HR): 0.531, 95% confidence interval (CI): 0.311–0.907, *p* = 0.018; Figure 5a), whereas non-significant differences in OS were observed in comparison to those with low relative abundance (median OS 19.9 vs. 11.4 months, HR: 0.642 (95% CI: 0.346–1.190), *p* = 0.155; Figure 5b). On the other hand, patients with high abundance of *Dialister* showed a significantly shortened PFS (median PFS 3.6 vs. 11.5 months, HR: 2.208 (95% CI: 1.286–3.791), *p* = 0.003; Figure 5c), and reduced OS compared to those with low abundance (median OS 9.3 vs. not reached months, HR: 2.847 (95% CI: 1.485–5.459), *p* = 0.001; Figure 5d).

We then performed multivariate analysis of the effect of *Phascolarctobacterium* and *Dialister* on PFS and OS, taking also into account other standard prognostic factors relevant in our cohort and the presence of irAEs. Our results demonstrated *Dialister* abundance as an independent prognostic factor for PFS (HR: 2.572 (95% CI: 1.352–4.895), *p* = 0.004) and OS (HR: 3.227 (95% CI: 1.574–6.613), *p* = 0.001) in advanced NSCLC patients (Figure 5e). Of note, multivariate analysis also revealed independent prognostic value for the presence of irAEs in our cohort (PFS HR: 0.299 (95% CI: 0.153–0.584), *p* < 0.001; OS HR: 0.414 (95% CI: 0.197–0.870), *p* = 0.020; Figure 5e), in agreement with a recent study that has evidenced association between multisystem irAEs and better outcomes in NSCLC [48]. Altogether, these results indicate that the low abundance of *Dialister* and the presence of irAEs might be used as prognostic biomarkers of improved survival for immunotherapy in NSCLC.

## 4. Discussion

In the present study, we prospectively investigated the gut microbiota profiles in 69 advanced NSCLC patients starting ICB monotherapy using 16S rDNA sequencing. Our results indicated that the ATB usage and ICB-related cutaneous toxicity, mainly skin rash, were associated with decreased gut microbiota diversity and differences in composition. Our findings also revealed differences in the gut microbiota composition in patients with clinical benefit (CB) versus those with progressive disease (PD). Concretely, *Phascolarctobacterium* was enriched in CB patients, and the high relative abundance of *Phascolarctobacterium* was associated with prolonged PFS. Contrarily, *Dialister* was overrepresented in PD patients and was identified as an independent prognostic biomarker.

Analysis of the sequencing data evidenced that *Bacteroides* was the most frequent genus in our cohort of advanced NSCLC patients. In agreement with these results, previous studies have also proved *Bacteroides* to be the most abundant genus in the human gut microbiota [49]. Furthermore, in lung cancer patients, *Bacteroides* is also significantly higher and a prominent genus biomarker compared to healthy controls [50].

It is widely accepted that the gut microbial composition can fluctuate in response to the use of ATB, affecting the abundances of up to 30% of the bacteria in the gut community [51]. Interestingly, some reports have demonstrated that the use of ATB is a negative factor in the effectiveness and clinical outcomes to ICB [29,33,34]. However, we revealed no significant relationship between ATB usage and the response to or survival from ICB immunotherapy. A plausible explanation lies in the resilience and the ability of the gut microbiota to adapt to the changing environment. It has been reported that the modification of the gut microbial community occurs within a few days from the first antibiotic dose and lasts for several weeks after treatment completion, with a remarkable ability of the gut microbiota to restore its compositional and functional state [52]. Although ATB use decreased the microbiota diversity at the baseline in our cohort, its composition might have recovered during the course of immunotherapy, resulting in non-significant association with clinical outcomes. Supporting our results, Huemer et al. neither observed the influence of ATB on ICB therapy in a bi-centric analysis of NSCLC patients [53]. Likewise, other studies have reported similar response rates and PFS for ICB medication in NSCLC patients treated with ATB and those not treated with ATB [32,54]. Nonetheless, despite the lack of relationship between the use of ATB and ICB efficacy in our patient cohort, and given the number of controversial results among studies, ATB prescription should still be recommended with caution both before and during immunotherapies. Indeed, several aspects, including the ATB administration route, type of ATB, time frame of ATB exposure prior to or during ICB treatment, as well as the interpatient heterogeneity, remain unclarified factors that might be associated with discrepant results among studies, thereby warranting further research to better define the impact of ATB usage on the outcomes of ICB immunotherapy.

The appearance of irAEs is an event that cannot be ignored in immunotherapy, since it may affect the management of cancer patients and could imply treatment discontinuation. The identification of factors predicting immune-related toxicity is a current need and preclinical and clinical studies have highlighted the impact of the gut microbiota on irAEs [28,40]. The gut microbiota has been proved to be a major regulator affecting skin homeostasis, likely through mechanisms related to the modulatory effects that the gut microbial community exerts on systemic immunity or via metabolite products, such as phenols that access circulation and get the distant skin [55,56]. In the present research, we have highlighted a decrease of the gut microbiota diversity in patients with skin toxicity. In our study, bacteria from the *Lactobacillales* and *Bifidobacteriales* orders were among the enriched bacteria in patients without ICB-related skin toxicity. Interestingly, *Lactobacillus* and *Bifidobacterium* are commonly tested probiotics that have demonstrated efficacy in the management of the dermatitis condition or chronic inflammatory diseases, such as psoriasis. In this sense, oral supplementation with *Lactobacilluscasei* in mouse models has been demonstrated to reduce skin inflammation by inhibiting IFN-γ signaling and CD8+ T hypersensitivity effector cells [57], as well as through increasing interleukin 10 (IL-10) secretion and activating FoxP3+ regulatory T cells in the skin [58]. On the other hand, in human subjects, administration of *Bifidobacterium* species has been proved to reduce systemic pro-inflammatory biomarkers, including C-reactive protein and tumor necrosis factor α (TNF-α) in psoriasis patients [59], and supplementation with a mix of *Bifidobacterium* and *Lactococcus* strains in pregnant women results in lower incidence of eczema in infants [60]. In the light of these studies, these probiotics might have potential reducing cutaneous toxicities and skin manifestations in ICB-treated patients. However, the bacteria species and mechanism through which the gut microbiota influences the appearance of skin toxicity need to be explored in future research.

Several studies have revealed that the gut microbiota may modulate the anti-tumor immune response, and certain gut microbiota compositions have been proved to be important regulatory factors in immunotherapy [28,31,35,36]. Our results also revealed differences in the gut microbiota composition according to response and clinical outcomes in advanced NSCLC patients treated with ICB. Concretely, high relative abundance of *Phascolarctobacterium* and low relative abundance of *Dialister*, two genera of the *Firmicutes* phylum, were significantly associated with better responses and improved survival from ICB treatment.

Supporting our findings, *Phascolarctobacterium* has been reported as one of the predominant bacteria in melanoma patients who responded to ICB [31]. Of note, one species of *Phascolarctobacterium* has been positively correlated with induction of colonic interferon-γ (IFN-γ)-expressing CD8 T cells, and colonization of mice with a consortium of 11 bacterial strains, including *Phascolarctobacterium faecium*, has been shown to enhance both spontaneous and ICB-mediated anti-tumor activity by increasing CD8+ tumor infiltrating lymphocytes producing IFN-γ in syngeneic tumor models [37]. On the other hand, some bacterial species that feed on dietary fibers produce metabolites such as SCFAs (mainly acetate, propionate, and butyrate) that exert positive effects on the large intestine mucosa, representing a primary energy source for colonocytes and maintaining intestinal homeostasis through anti-inflammatory actions. In this regard, *Phascolarctobacterium* is a good producer of the SCFAs propionate and acetate [61]. SCFAs are known to modulate immune cell function with implications in anti-cancer immunotherapies. Indeed, it has been demonstrated that higher concentrations of fecal SCFAs are associated with ICB efficacy in solid cancer tumors [38] and predict long-term beneficial effects in NSCLC patients [62]. Thus, we hypothesize that *Phascolarctobacterium* might predict clinical benefit in our cohort by increasing the levels of SCFAs and modulating anti-tumor immune responses.

On the other hand, *Dialister* is a genus classified within the family *Veillonellaceae*, and several reports have demonstrated the pathogenic potential of *Dialister* spp. In this sense, *Dialister* has been associated with periodontitis, and decreased levels of *Dialister* spp. have been observed in patients with Crohn’s disease [63,64]. Regarding cancer, this microorganism has been found to be increased in microbiota samples of head and neck squamous cell carcinoma and in NSCLC patients in comparison to healthy individuals [65,66]. A recent study has estimated the metabolic capacity of *Dialister* to produce the SCFA pentanoate, also known as valerate [67]. It has been reported that pentanoate has a marked immunomodulatory effect by inducing the production of the anti-inflammatory cytokine IL-10 and suppressing Th17 cells, therefore showing therapeutic potential in inflammatory diseases [68]. Although the specific mechanism through which *Dialister* undermines the efficacy of ICB warrants further investigation, its anti-inflammatory effects might explain the detrimental role that this microorganism displays in NSCLC patients receiving immunotherapy.

## 5. Conclusions

This study corroborates the predictive/prognostic significance of the baseline gut microbiota composition in advanced NSCLC patients treated with ICB. Our data suggest that the use of ATB is associated with decreased microbiota diversity, but does not influence the therapeutic efficacy of ICB in our patient cohort. Low microbiota diversity is correlated with development of ICB-related skin toxicity, and the appearance of irAEs is an independent prognostic biomarker for improved survival. In addition, the gut microbiota composition reveals *Phascolarctobacterium* and *Dialister* as potent predictive and prognostic factors. *Phascolarctobacterium* was correlated with clinical benefit and prolonged progression-free survival, whereas *Dialister* was associated with progressive disease and reduced progression-free survival and overall survival. If proven in larger cohorts, these microorganisms might be applied as potential biomarkers for ICB treatment in NSCLC. Moreover, subsequent extensive cohorts and clinical trials could analyze the possibility of combining *Phascolarctobacterium* and/or *Dialister* with other validated biomarkers, such as PD-L1 expression or tumor mutational burden, in order to provide powerful predictive/prognostic signatures for immunotherapy in NSCLC. Finally, if confirmed, our findings raise the need for further mechanistic studies to determine the biological relevance of *Phascolarctobacterium* and *Dialister* in the modulation of anti-tumor immune responses.

## Figures and Tables

**Table 1:** Baseline clinico-pathological characteristics of patients and correlation with response to ICB.

Characteristics	Total (*n* = 69)	CB (*n* = 33)	PD (*n* = 36)	*p*-Value
Age				0.276
≤66	34 (49.3%)	14 (42.4%)	20 (55.6%)	
>66	35 (50.7%)	19 (57.6%)	16 (44.4%)	
Gender				**0.015**
Male	49 (71.0%)	28 (84.8%)	21 (58.3%)	
Female	20 (29.0%)	5 (15.2%)	15 (41.7%)	
Smoking status				0.805
Smoker	47 (68.1%)	22 (66.7%)	25 (69.4%)	
Non-smoker	22 (31.9%)	11 (33.3%)	11 (30.6%)	
Body mass index				**0.035**
High (≥25)	32 (46.4%)	20 (60.6%)	12 (33.3%)	
Low (<25)	33 (47.8%)	12 (36.4%)	21 (58.3%)	
Non-specified	4 (5.8%)	1 (3.0%)	3 (8.4%)	
Antibiotics previous				0.710
Yes	16 (23.2%)	7 (21.2%)	9 (25.0%)	
No	53 (76.8%)	26 (78.8%)	27 (75.0%)	
ECOG performance status				0.110
0–1	63 (91.3%)	32 (97.0%)	31 (86.1%)	
≥2	6 (8.7%)	1 (3.0%)	5 (13.9%)	
Stage				0.151
III	12 (17.4%)	8 (24.2%)	4 (11.1%)	
IV	57 (82.6%)	25 (75.8%)	32 (88.9%)	
Histology				0.528
Adenocarcinoma	32 (46.4%)	14 (42.4%)	18 (50.0%)	
Non-adenocarcinoma	37 (53.6%)	19 (57.6%)	18 (50.0%)	
PD-L1				**0.034**
Positive (TPS ≥ 1%)	46 (66.7%)	26 (78.8%)	20 (55.6%)	
Negative (TPS < 1%)	21 (30.4%)	6 (18.2%)	15 (41.7%)	
Non-specified	2 (2.9%)	1 (3.0%)	1 (2.8%)	
Immunotherapy				0.265
1st line	37 (53.6%)	20 (60.6%)	17 (47.2%)	
Non-1st line	32 (46.4%)	13 (39.4%)	19 (52.8%)	
Tumor size				0.180
≤5 cm	16 (23.2%)	10 (30.3%)	6 (16.7%)	
>5 cm	53 (76.8%)	23 (69.7%)	30 (83.3%)	
Number of metastatic sites				0.127
<2	27 (39.1%)	16 (48.5%)	11 (30.6%)	
≥2	42 (60.9%)	17 (51.5%)	25 (69.4%)	

Non-smoker: never smoker + former smoker; ECOG: Eastern cooperative oncology group; TPS: tumor proportion score. *p*-values were obtained using the chi-square test. Bold *p*-values denote statistical significance at the *p <* 0.05 level.

## Data Availability

The data presented in this study are available in this article and attached supplementary files.

## Supplementary Materials

The following are available online at https://www.mdpi.com/article/10.3390/cancers13112514/s1: Figure S1: Correlation analysis between immune-related adverse events and clinical outcomes; Figure S2: Correlation analysis between alpha-diversity and immune-related adverse events; Figure S3: Correlation analysis between alpha-diversity and clinical outcomes; Figure S4: UpSet plots illustrating quantitative intersection of the exclusive taxa for CB (a) and PD (b) patients; Figure S5: Principal component analysis (PCA) plot of the gut microbiota composition in the CB and PD groups; Table S1: Detailed clinico-pathological characteristics of 69 advanced NSCLC patients in the study; Table S2: Univariate analysis based on clinico-pathological characteristics; Table S3: Sequencing information and alpha-diversity indices of 69 advanced NSCLC patients; Table S4: Correlation analysis between alpha-diversity and clinico-pathological characteristics.
