# Supplementary material for: Analysis of the Gut Microbiota: An Emerging Source of Biomarkers for Immune Checkpoint Blockade Therapy in Non-Small Cell Lung Cancer
Source: Cancers (Basel). 2021 May 21;13(11):2514. doi: 10.3390/cancers13112514 (PMC8196639; doi:10.3390/cancers13112514)
Supplement: Supplementary file 1 [file cancers-13-02514-s001.zip › Figure S1.pdf]

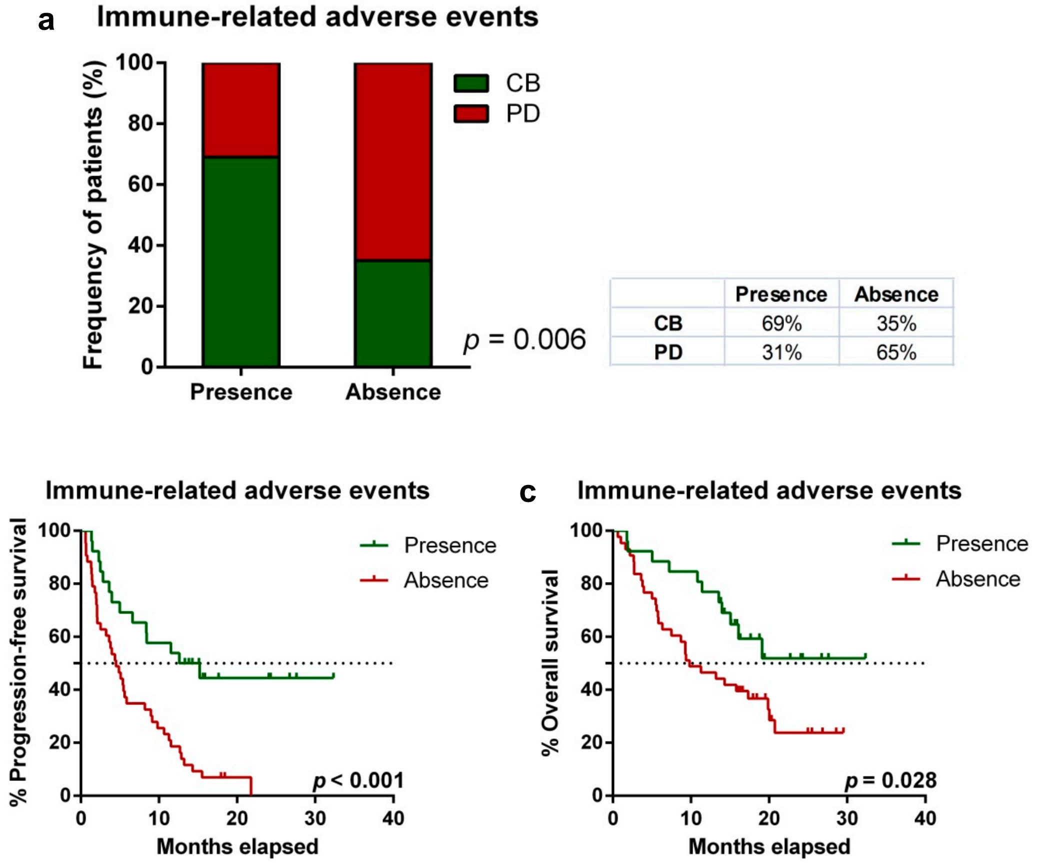

	Presence	Absence
CB	69%	35%
PD	31%	65%

Figure S1. Correlation analysis between immune-related adverse events and clinical outcomes. (**a**) Frequency of patients stratified by presence/absence of immune-related adverse events according to response to ICB (CB and PD groups). Statistical analysis was performed using the Chi-squared test. (**b**) Kaplan-Meier plot of progression-free survival in patients stratified by presence/absence of immune-related adverse events. (**c**) Kaplan-Meier plot of overall survival in patients stratified by presence/absence of immune-related adverse events. CB: clinical benefit group; PD: progression disease group. Statistical analysis was performed using the log-rank test.
