# Supplementary material for: Analysis of the Gut Microbiota: An Emerging Source of Biomarkers for Immune Checkpoint Blockade Therapy in Non-Small Cell Lung Cancer
Source: Cancers (Basel). 2021 May 21;13(11):2514. doi: 10.3390/cancers13112514 (PMC8196639; doi:10.3390/cancers13112514)
Supplement: Supplementary file 1 [file cancers-13-02514-s001.zip › Figure S2.pdf]

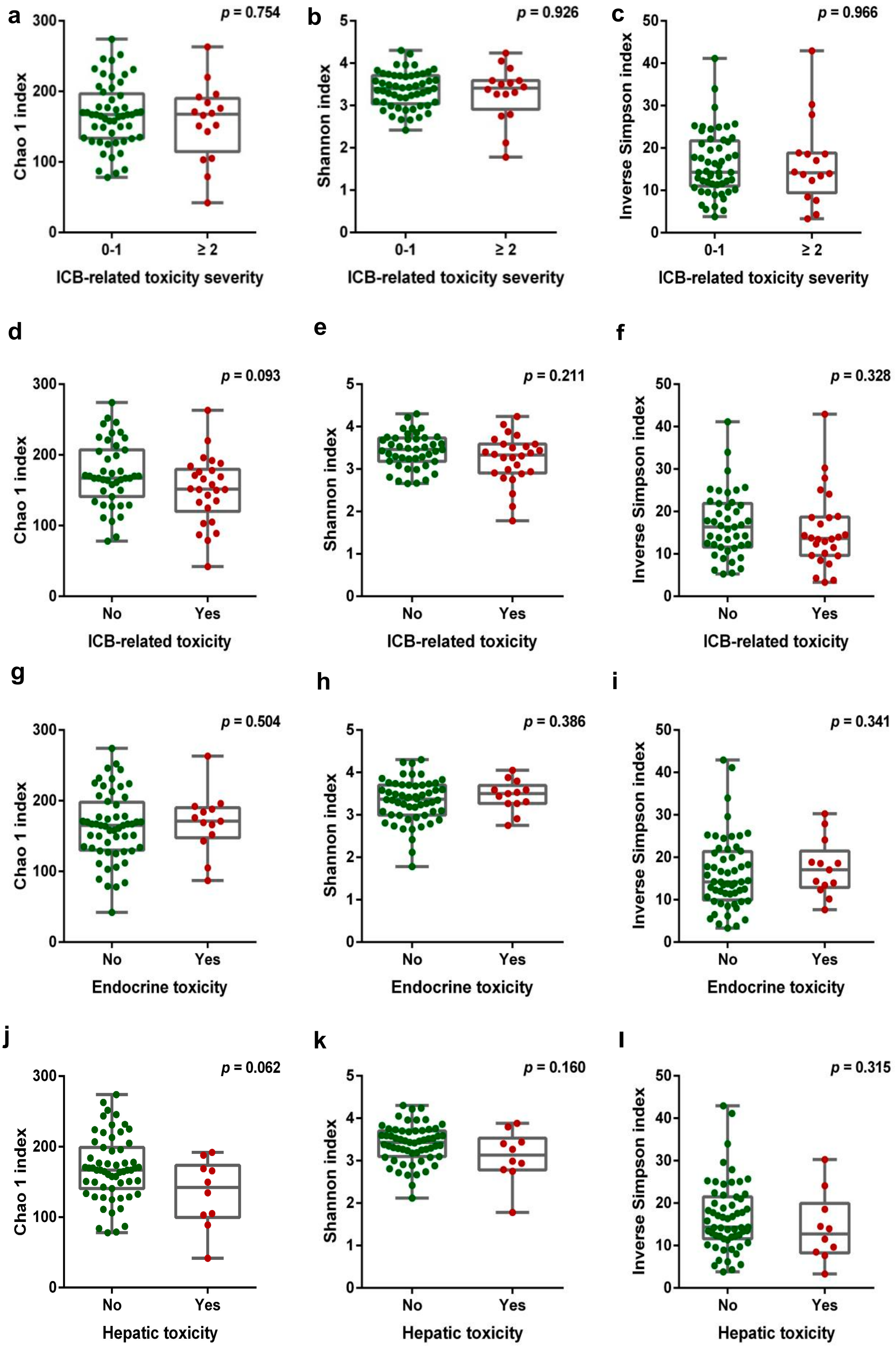
a
p = 0.754
Chao 1 index
ICB-related toxicity severity
0-1
≥ 2
b
p = 0.926
Shannon index
ICB-related toxicity severity
c
p = 0.966
Inverse Simpson index
ICB-related toxicity severity
d
p = 0.093
Chao 1 index
ICB-related toxicity
No
Yes
e
p = 0.211
Shannon index
ICB-related toxicity
f
p = 0.328
Inverse Simpson index
ICB-related toxicity
g
p = 0.504
Chao 1 index
Endocrine toxicity
h
p = 0.386
Shannon index
Endocrine toxicity
i
p = 0.341
Inverse Simpson index
Endocrine toxicity
j
p = 0.062
Chao 1 index
Hepatic toxicity
k
p = 0.160
Shannon index
Hepatic toxicity
l
p = 0.315
Inverse Simpson index
Hepatic toxicity

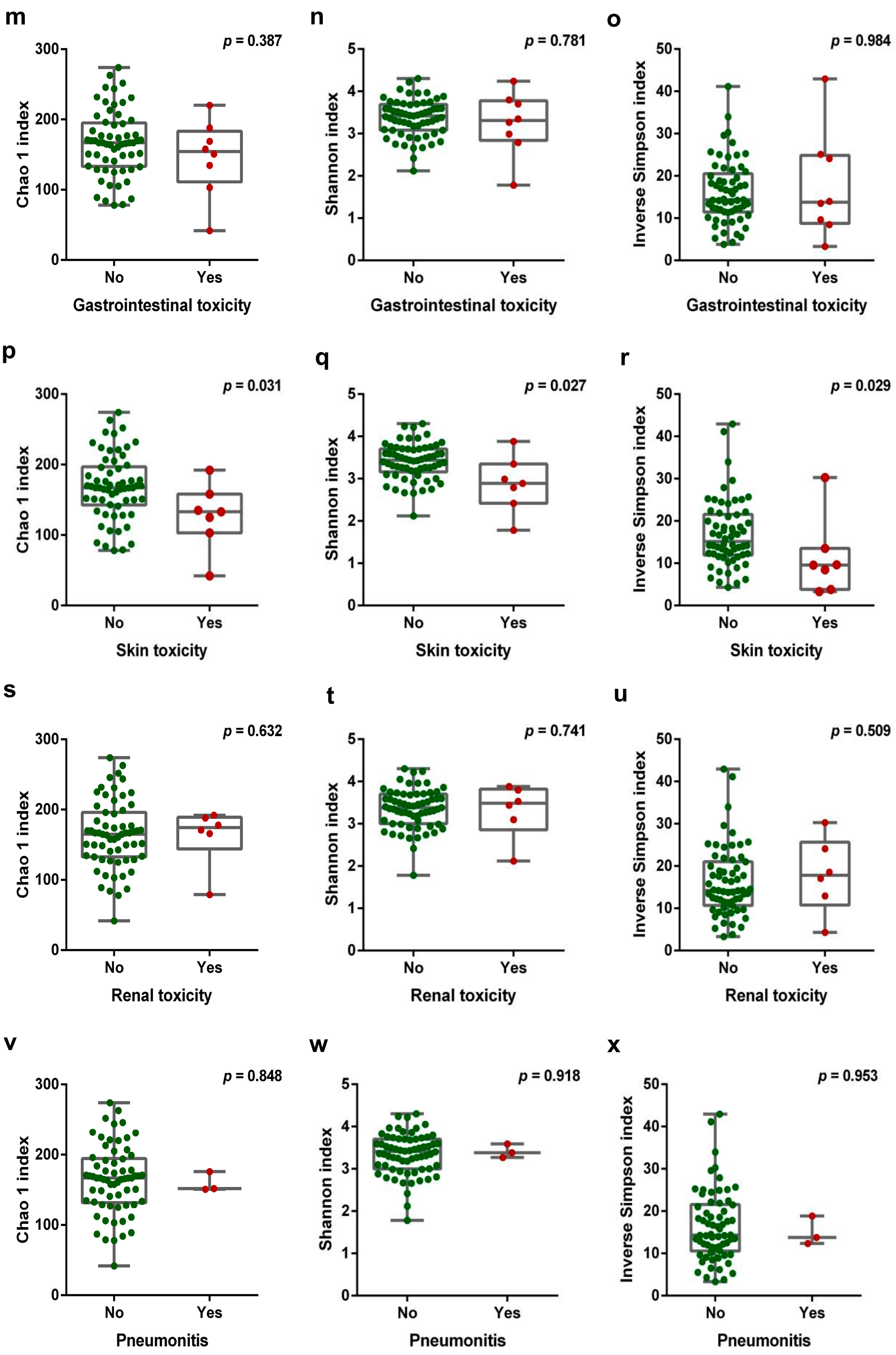
m
p = 0.387
Chao 1 index
0
100
200
300
No
Yes
Gastrointestinal toxicity
n
p = 0.781
Shannon index
0
1
2
3
4
5
No
Yes
Gastrointestinal toxicity
o
p = 0.984
Inverse Simpson index
0
10
20
30
40
50
No
Yes
Gastrointestinal toxicity
p
p = 0.031
Chao 1 index
No
Yes
Skin toxicity
q
p = 0.027
Shannon index
No
Yes
Skin toxicity
r
p = 0.029
Inverse Simpson index
No
Yes
Skin toxicity
s
p = 0.632
Chao 1 index
No
Yes
Renal toxicity
t
p = 0.741
Shannon index
No
Yes
Renal toxicity
u
p = 0.509
Inverse Simpson index
No
Yes
Renal toxicity
v
p = 0.848
Chao 1 index
No
Yes
Pneumonitis
w
p = 0.918
Shannon index
No
Yes
Pneumonitis
x
p = 0.953
Inverse Simpson index
No
Yes
Pneumonitis

Figure S2. Correlation analysis between alpha-diversity and immune-related adverse events. (**a**-**c**) Alpha-diversity indices scores of the gut microbiota in patients stratified according to immune checkpoint blockade (ICB)-related toxicity severity. (**d**-**f**) Alpha-diversity indices scores of the gut microbiota in patients stratified by presence/absence of ICB-related toxicity. (**g**-**i**) Alpha-diversity indices scores of the gut microbiota in patients stratified by presence/absence of endocrine toxicity. (**j**-**l**) Alpha-diversity indices scores of the gut microbiota in patients stratified by presence/absence of hepatic toxicity. (**m**-**o**) Alpha-diversity indices scores of the gut microbiota in patients stratified by presence/absence of gastrointestinal toxicity. (**p**-**r**) Alpha-diversity indices scores of the gut microbiota in patients stratified by presence/absence of skin toxicity. (**s**-**u**) Alpha-diversity indices scores of the gut microbiota in patients stratified by presence/absence of renal toxicity. (**v**-**x**) Alpha-diversity indices scores of the gut microbiota in patients stratified by presence/absence of pulmonary toxicity (pneumonitis). Statistical analysis was performed using the Mann-Whitney *U* rank sum test. Error bars represent the distribution of alpha-diversity scores.
