# Supplementary material for: Analysis of the Gut Microbiota: An Emerging Source of Biomarkers for Immune Checkpoint Blockade Therapy in Non-Small Cell Lung Cancer
Source: Cancers (Basel). 2021 May 21;13(11):2514. doi: 10.3390/cancers13112514 (PMC8196639; doi:10.3390/cancers13112514)
Supplement: Supplementary file 1 [file cancers-13-02514-s001.zip › Figure S3.pdf]

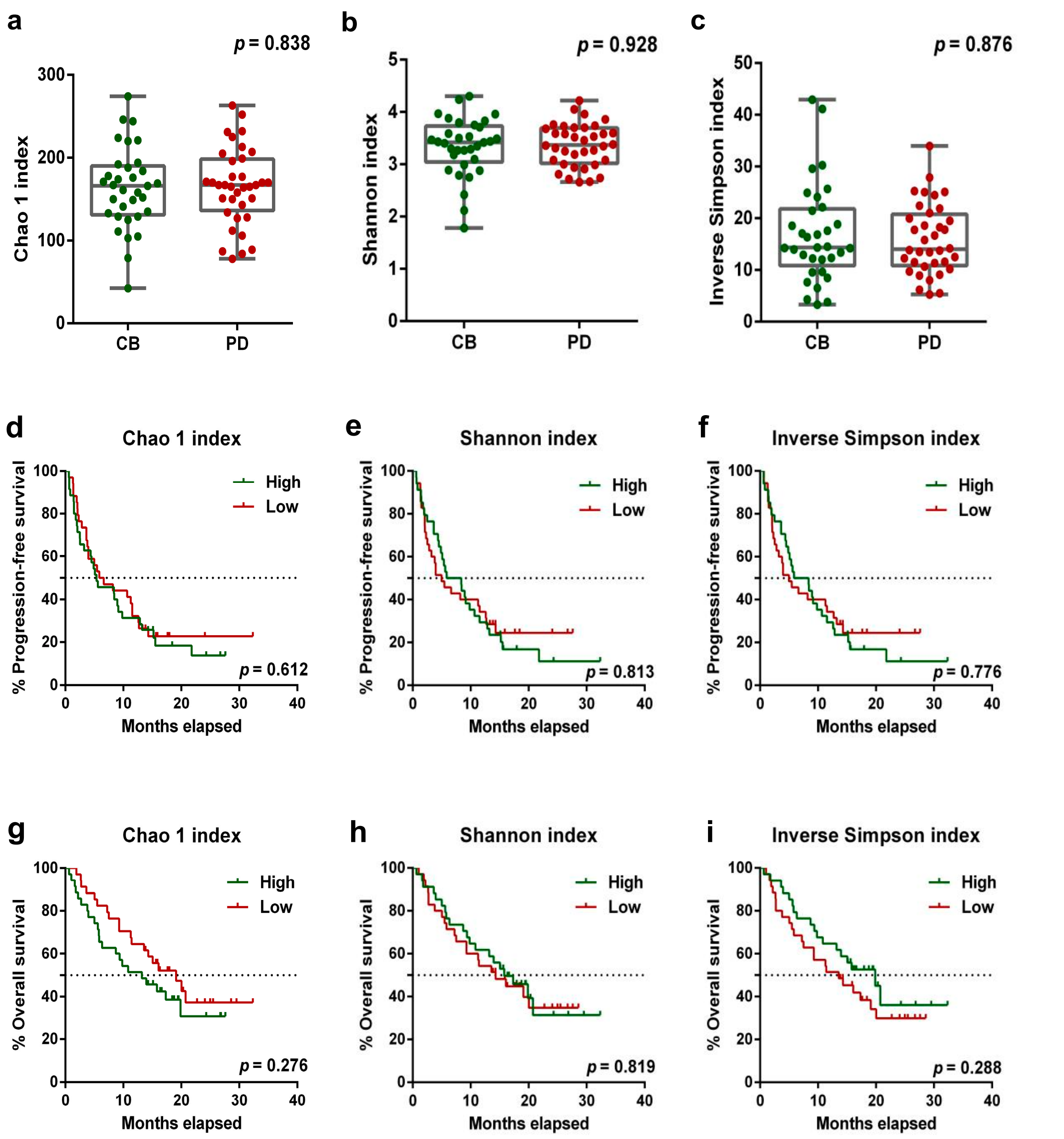

Figure S3. Correlation analysis between alpha-diversity and clinical outcomes. (**a**) Chao 1, (**b**) Shannon and (**c**) inverse Simpson indices scores of the gut microbiota in patients stratified according to response to ICB. CB: clinical benefit; PD: progression disease. Statistical analysis was performed using the Mann-Whitney *U* rank sum test. Error bars represent the distribution of alpha-diversity scores. (**d-f**) Kaplan-Meier plot of progression-free survival in patients with high or low alpha-diversity indices. (**g-i**) Kaplan-Meier plot of overall survival in patients with high or low alpha-diversity indices. Cutoff values correspond to the median of index scores: 167 (Chao 1 index), 3.4

(Shannon index) and 14.25 (inverse Simpson index). Statistical analysis was performed using the log-rank test.
