# Supplementary material for: Analysis of the Gut Microbiota: An Emerging Source of Biomarkers for Immune Checkpoint Blockade Therapy in Non-Small Cell Lung Cancer
Source: Cancers (Basel). 2021 May 21;13(11):2514. doi: 10.3390/cancers13112514 (PMC8196639; doi:10.3390/cancers13112514)
Supplement: Supplementary file 1 [file cancers-13-02514-s001.zip › Figure S4.pdf]

a

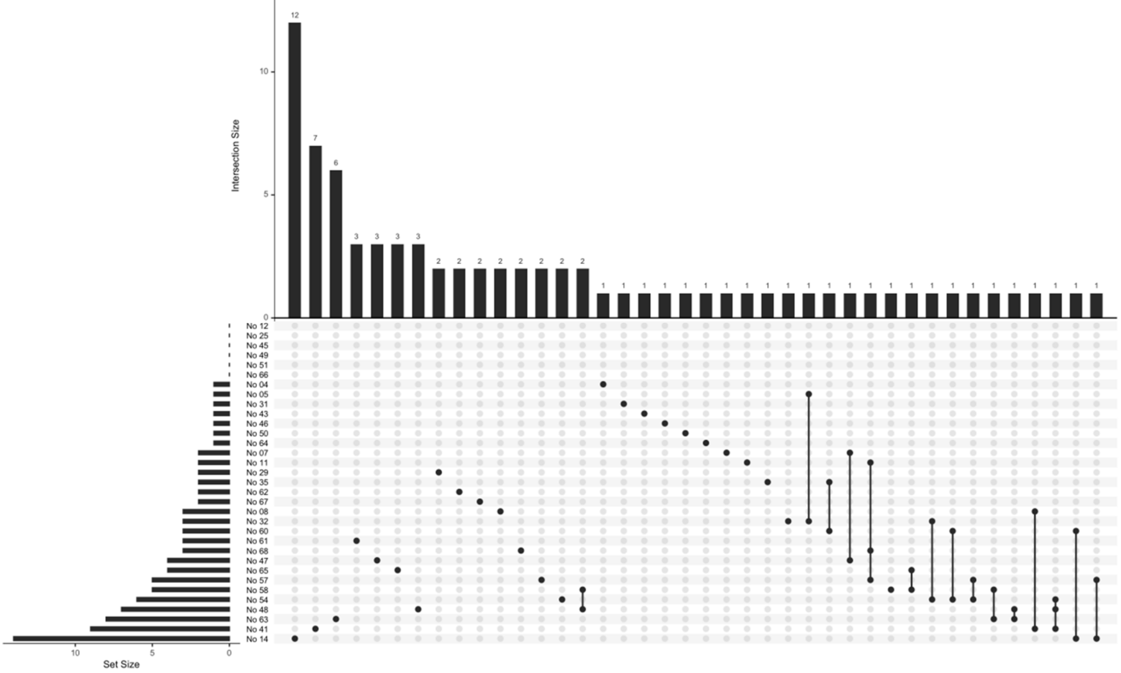

b

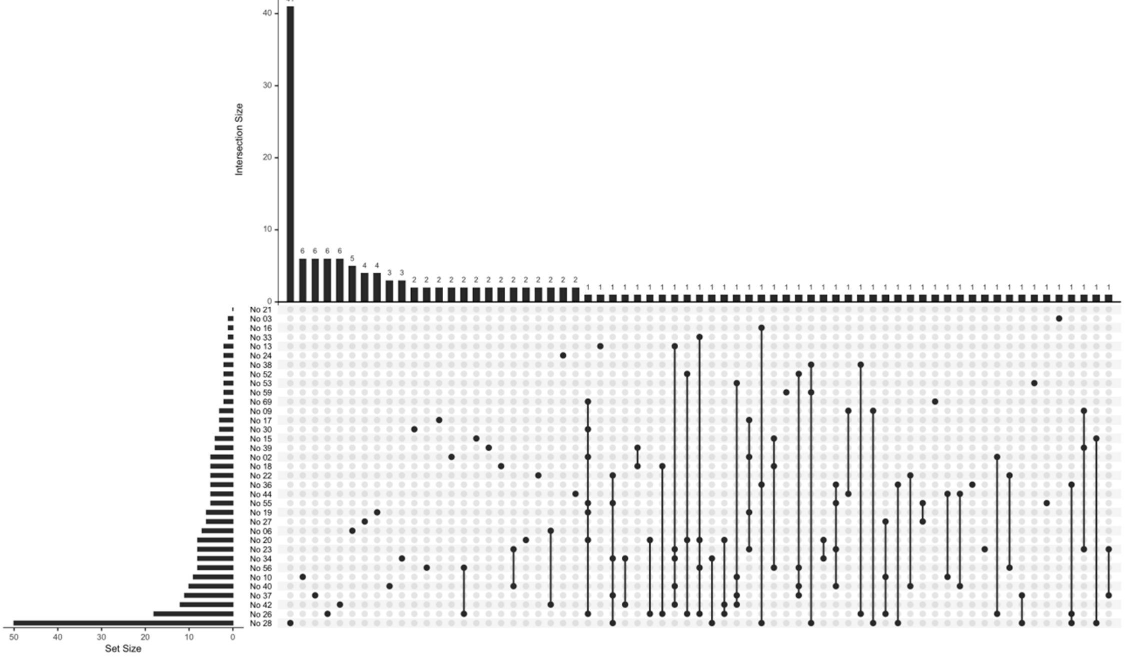

Figure S4. UpSet plots illustrating quantitative intersection of the exclusive taxa for CB (a) and PD (b) patients. The numbers above the bars show the number of common taxa between the groups of samples marked below the bars. Empty (light-gray) circles indicate samples without detected taxa, dark circles indicate samples with detected taxa and connecting bar

indicate multiple overlapping samples. CB: clinical benefit group; PD: progression disease group.
