# Supplementary material for: Analysis of the Gut Microbiota: An Emerging Source of Biomarkers for Immune Checkpoint Blockade Therapy in Non-Small Cell Lung Cancer
Source: Cancers (Basel). 2021 May 21;13(11):2514. doi: 10.3390/cancers13112514 (PMC8196639; doi:10.3390/cancers13112514)
Supplement: Supplementary file 1 [file cancers-13-02514-s001.zip › Figure S5.pdf]

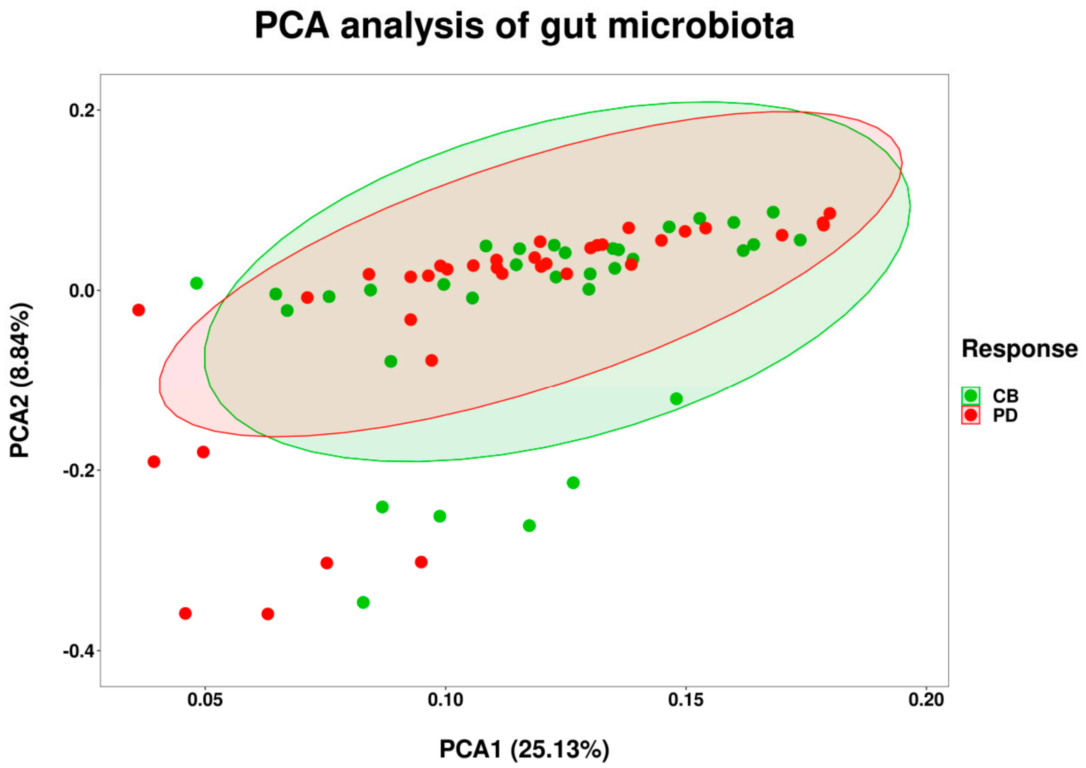

Figure S5. Principal component analysis (PCA) plot of the gut microbiota composition in CB and PD groups. PERMANOVA with ''ADONIS'' was performed to investigate the significance between the tested groups, $p = 0.184$. CB (green): clinical benefit group; PD (red): progression disease group.
